# Developing a Glyoxal-Crosslinked Chitosan/Gelatin Hydrogel for Sustained Release of Human Platelet Lysate to Promote Tissue Regeneration

**DOI:** 10.3390/ijms22126451

**Published:** 2021-06-16

**Authors:** Ching-Cheng Tsai, Tai-Horng Young, Guang-Shih Chen, Nai-Chen Cheng

**Affiliations:** 1 Department of Surgery, National Taiwan University Hospital and College of Medicine, Taipei 100, Taiwan; d9876212@gmail.com; 2 Institute of Biomedical Engineering, College of Medicine and College of Engineering, National Taiwan University, Taipei 100, Taiwan; thyoung@ntu.edu.tw (T.-H.Y.); gary3217173@gmail.com (G.-S.C.)

**Keywords:** human platelet lysate, chitosan, gelatin, glyoxal, hydrogel, angiogenesis, tissue regeneration

## Abstract

The clinical application of human platelet lysate (HPL) holds promise for tissue regeneration, and the development of an efficient vehicle for its delivery is desired. Chitosan-based hydrogels are potential candidates, but they often exhibit weak mechanical properties. In this study, a chitosan/gelatin (CS-GE) hydrogel crosslinked by glyoxal was fabricated for sustained release of HPL. The influence of HPL on Hs68 fibroblast and human umbilical vein endothelial cell (HUVEC) culture was evaluated, and we found that supplementing 5% HPL in the medium could significantly improve cell proliferation relative to supplementing 10% fetal bovine serum (FBS). Moreover, HPL accelerated the in vitro wound closure of Hs68 cells and facilitated the tube formation of HUVECs. Subsequently, we fabricated CS-GE hydrogels crosslinked with different concentrations of glyoxal, and the release pattern of FITC-dextrans (4, 40 and 500 kDa) from the hydrogels was assessed. After an ideal glyoxal concentration was determined, we further characterized the crosslinked CS-GE hydrogels encapsulated with different amounts of HPL. The HPL-incorporated hydrogel was shown to significantly promote the proliferation of Hs68 cells and the migration of HUVECs. Moreover, the release pattern of transforming growth factor-β1 (TGF-β1) and platelet-derived growth factor-BB (PDGF-BB) from hydrogel was examined in vitro, demonstrating a sustained release profile of the growth factors. Finally, the chick chorioallantoic membrane assay revealed that HPL encapsulation in the hydrogel significantly stimulated angiogenesis in ovo. These results demonstrate the great potential of the crosslinked CS-GE hydrogel to serve as an effective delivery system for HPL to promote tissue regeneration.

## 1. Introduction

Abundant growth factors and cytokines are stored in platelet granules, and they can be naturally released by thrombin activation and clotting or artificially released by freeze/thaw-mediated platelet lysis, sonication or chemical treatment [1]. Clinically, these platelet concentrates have been widely applied in a variety of fields, such as the treatment of symptomatic oral lichen planus [2], lateral epicondylitis [3] and medication-related osteonecrosis of the jaws [4]. Although the efficacy of platelet concentrates in promoting wound healing and tissue regeneration is at the center of a recent academic debate, different formats of platelet concentrates have been prepared to test their regenerative effects.

Human platelet lysate (HPL), a biological material derived from platelet concentrates, can be manufactured as a cost-effective and standardized cell culture supplement [5,6]. HPL is known to contain abundant mitogenic growth factors, including vascular endothelial growth factor (VEGF), basic fibroblast growth factor (bFGF), epidermal growth factor (EGF), platelet-derived growth factors (PDGF) and transforming growth factor (TGF) [7,8]. These growth factors not only exert a positive effect on angiogenesis and post-ischemic vascular remodeling but also play a critical role in tissue inflammation and regeneration [9,10,11]. Therefore, platelet lysates have demonstrated the advantages of replacing fetal bovine serum (FBS) for culturing various types of cells, including stem cells, fibroblasts and endothelial cells [1,12]. The use of HPL in cell culture is desired for clinical use by circumventing the risks of xeno-immunization against bovine antigens and the transmission of zoonotic diseases [13,14]. Moreover, the effect of HPL on directly promoting angiogenesis and tissue regeneration has been demonstrated [15,16], so HPL holds great promise for treating various tissue defects.

The development of efficient biomaterials to assist HPL delivery for tissue regeneration remains a challenge. To facilitate a sustained release of its contents in the defect site, HPL could be blended with biomaterials, adsorbed onto scaffolds or used as a gel to mediate neo-angiogenesis [17,18,19]. However, the currently available options are not ideal, and a novel hydrogel system is desired to allow sustained HPL delivery. Hydrogel is a polymer with a large amount of water but is not soluble in water. It is usually soft and flexible with good fluid-retaining capability and biocompatibility, so it has been widely applied in various biomedical fields as a kind of drug delivery system [20,21]. Chitosan-based thermosensitive hydrogels, fabricated with a chitosan/β-glycerophosphate (β-GP) system, have been successfully applied in drug release studies [22], and they were further applied to deliver cells for tissue engineering purposes [23,24]. β-GP acts as a proton receiver from the positively charged chitosan at elevated temperature, thereby inducing chitosan gelation [25]. Moreover, our previous study demonstrated superior biocompatibility when gelatin was blended into this chitosan/β-GP system [26].

When gelatin and chitosan are mixed for hydrogel fabrication, they form poly-electrolytic complexes in different gelated states, and the structure mimics the natural components of the extracellular matrix [27,28]. However, the mechanical property of the composite hydrogel is still not ideal. To achieve better mechanical strength and a controlled release pattern, glyoxal was selected as a crosslinker to reinforce the hydrogel in this study [29,30]. Glyoxal is the smallest dialdehyde with high water solubility, and it exhibits lower cytotoxicity when compared to most chemical crosslinkers [29,30,31]. It was expected to crosslink the available amine groups on chitosan and gelatin chains to form the network [32], rendering improved mechanical properties of the chitosan/gelatin (CS-GE) hydrogel. In this study, we aimed to fabricate a CS-GE hydrogel crosslinked by glyoxal for the sustained release of HPL. An ideal concentration of glyoxal was selected, resulting in negligible cytotoxicity with a reasonable level of mechanical strength. These characteristics can facilitate efficient HPL delivery from the crosslinked CS-GE hydrogel to promote angiogenesis and regeneration in damaged tissues.

## 2. Results

### 2.1. HPL Enhanced the Proliferation and Migration of Hs68 Fibroblasts

**Figure 1:**
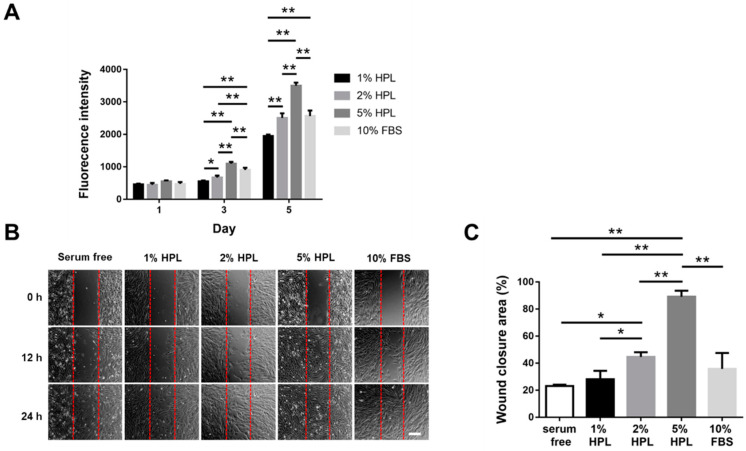
The influence of different concentrations of HPL on the proliferation and migration of Hs68 fibroblasts. (**A**) Alamar Blue assay revealed the proliferative activity of Hs68 fibroblasts incubated in different concentrations of HPL or 10% FBS. (**B**) The representative images of in vitro cell migration at 0, 12 and 24 h (scale bar = 200 μm). (**C**) Quantification of the wound closure percentage of Hs68 fibroblasts at 12 h. Serum-free was used as the control group. (* *p* < 0.05, ** *p* < 0.01).

In the result of the Alamar Blue assay, a higher concentration of HPL in the medium resulted in higher fluorescence intensity, suggesting a higher proliferative activity. The 5% HPL group even demonstrated a significantly higher value of fluorescence intensity on day 5 than the 10% FBS group (3498.0 ± 96.0 vs. 2560.0 ± 175.0, *p* < 0.01; Figure 1A). Moreover, fibroblast migration was assessed to determine whether HPL can accelerate the process of wound healing. After Hs68 cells were cultured in different conditions for 12 h, the images showed that the cell coverage area in the experimental group, 5% HPL, was higher than that in the other experimental groups. At 24 h, the gap was nearly filled by the migrant cells in the 5% HPL group, while some cell-free zones could still be observed in other groups (Figure 1B). ImageJ software was used to quantify the wound closure area at the time point of 12 h, revealing a significantly higher wound closure area in the 5% HPL group relative to the 10% FBS group (89.2% ± 7.6% vs. 35.8% ± 20.3%, *p* < 0.01; Figure 1C).

### 2.2. HPL Enhanced the Proliferation and Tube Formation of Endothelial Cells

**Figure 2:**
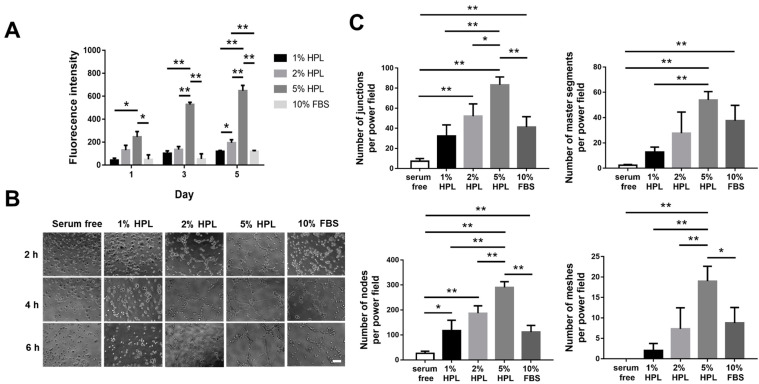
The influence of different concentrations of HPL on the proliferation and tube formation of HUVECs. (**A**) Alamar Blue assay revealed the proliferative activity of HUVECs incubated in different concentrations of HPL or 10% FBS. (**B**) The representative images of endothelial cell tube formation under different conditions at 2, 4 and 6 h (scale bar = 100 μm). (**C**) The number of junctions, master segments, nodes and meshes at 4 h was compared among different groups. Serum-free was used as the control group. (* *p* < 0.05, ** *p* < 0.01).

In the proliferation assay of human umbilical vein endothelial cells (HUVECs), the 5% HPL group on day 5 displayed a significantly higher proliferative activity than the other groups (fluorescence intensity: 5% HPL: 647.7 ± 46.8 vs. 1% HPL: 119.0 ± 8.5, 2% HPL: 194.7 ± 26.5 and 10% FBS: 118.7 ± 8.5, *p* < 0.01, respectively; Figure 2A). Furthermore, we examined the tube formation of HUVECs under different culture conditions at different time points (Figure 2B). ImageJ software was used to quantify the cell images at 4 h. The 5% HPL group exhibited significantly more tube-like structures than the 10% FBS group (83.3 ± 7.8 vs. 41.3 ± 10.2 for junctions, *p* < 0.01; 290.0 ± 23.1 vs. 111.8 ± 26.4 for nodes, *p* < 0.01; 19.0 ± 3.6 vs. 17.4 ± 2.9 for meshes, *p* < 0.05; Figure 2C).

### 2.3. Characterization of CS-GE Hydrogel

To fabricate the designated HPL carrier, CS-GE hydrogels were crosslinked with different concentrations of glyoxal, and their biomechanical properties were assessed. The gelation time of CS-GE hydrogels was determined by the vial tilting method, which showed a shorter gelation time at a higher glyoxal concentration (Table 1).

**Figure 3:**
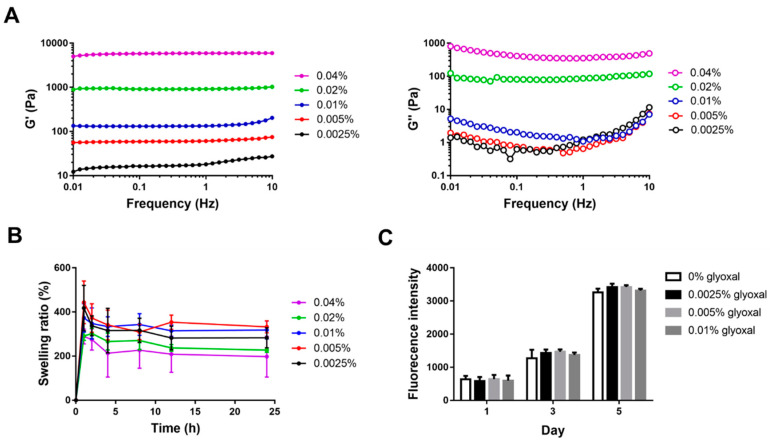
The mechanical properties and cytotoxic effects of CS-GE hydrogel crosslinked with different glyoxal concentrations. (**A**) In a frequency sweep mode, the viscoelastic properties of the hydrogels, including the storage modulus (G′) and the loss modulus (G′′), were analyzed. (**B**) Swelling ratio of the hydrogels. (**C**) Cytotoxicity of different concentrations of glyoxal in the medium was tested by the Alamar Blue assay to reveal the viability of Hs68 fibroblasts.

The viscoelastic properties of CS-GE hydrogels were analyzed by a rheometer. The shear storage modulus (G′, reflecting the elastic behavior) and shear loss modulus (G′′, reflecting the viscous behavior) of the hydrogels were characterized. At a higher concentration of glyoxal, a higher value of G′ was noted, with the 0.04% glyoxal group possessing the highest G′ (nearly 6000 Pa) compared to the other groups. The 0.04% glyoxal group also exhibited the highest value of G′′ (nearly 400 Pa), ranging from 0.1 to 10 Hz (Figure 3A).

These results revealed that a relatively higher glyoxal concentration is essential for the fabrication of hydrogels with greater mechanical strength.

The swelling property of crosslinked CS-GE hydrogels was investigated by measuring the changes of hydrogel weight during incubation in phosphate-buffered saline (PBS). After 1 h of incubation, all CS-GE hydrogels reached a maximal swelling ratio, displaying 417.7% ± 103.3%, 443.5% ± 96.7%, 373.3% ± 64.6%, 290.9% ± 19.0% and 288.8% ± 33.7% (from 0.0025% to 0.04% glyoxal group), respectively. After that time point, the swelling ratio gradually decreased in all groups, displaying 283.5% ± 45.6%, 332.6% ± 27.3%, 318.4% ± 40.6%, 227.3% ± 19.0% and 288.8% ± 33.7% after 24 h of incubation (Figure 3B).

### 2.4. In Vitro Cytotoxicity Assay of Glyoxal

To evaluate the potential cytotoxicity of glyoxal, the influence of unreacted glyoxal in the cell culture medium on the cellular viability of Hs68 fibroblasts was tested by Alamar Blue assay. No significant difference among all groups was detected during incubation for 5 days (Figure 3C), suggesting negligible cytotoxicity if the concentration of glyoxal in the medium was less than 0.01%.

### 2.5. Scanning Electron Micrographs and Pore Size Measurement

**Figure 4:**
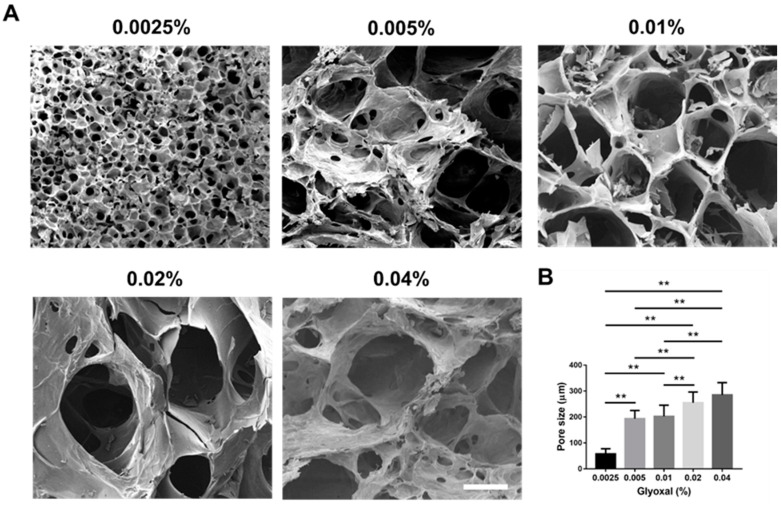
The porosity of CS-GE hydrogels crosslinked with different glyoxal concentrations. (**A**) The representative scanning electron micrographs of lyophilized CS-GE hydrogels crosslinked with different concentrations of glyoxal (scale bar = 200 μm). (**B**) Pore size measurement revealed significant differences among different CS-GE hydrogels. (** *p* < 0.01).

Scanning electron microscope (SEM) images showed the morphology of lyophilized hydrogel crosslinked with different concentrations of glyoxal, and all groups presented with high porous structures (Figure 4A). For the pore size measurement, using a higher concentration of glyoxal resulted in a larger pore size of the hydrogel. The 0.0025% glyoxal group possessed a significantly smaller pore size than the other groups (0.0025% glyoxal: 57.8 ± 19.7 μm vs. 0.005% glyoxal: 193.9 ± 31.3 μm, 0.01% glyoxal: 202.4 ± 43.2 μm, 0.02% glyoxal: 254.8 ± 41.8 μm and 0.04% glyoxal: 285.2 ± 47.6 μm, *p* < 0.01, respectively; Figure 4B).

### 2.6. Enzymatic Degradation Assay

Although a higher glyoxal concentration for hydrogel crosslinking resulted in stronger mechanical properties, the shorter gelation time (less than 20 s) and higher viscosity may render it difficult to apply or inject. Hence, we chose the 0.0025%, 0.005% and 0.01% glyoxal groups to further conduct the hydrogel degradation and drug release experiments.

**Figure 5:**
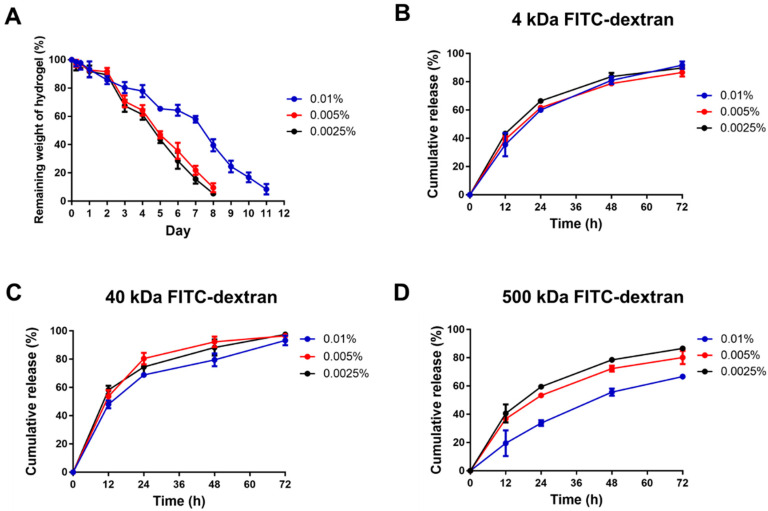
The degradation of CS-GE hydrogels and the release pattern of encapsulated FITC-dextrans. (**A**) In vitro degradation test of CS-GE hydrogel crosslinked with different concentrations of glyoxal. The release percentage of (**B**) 4 kDa, (**C**) 40 kDa and (**D**) 500 kDa FITC-dextrans from CS-GE hydrogels crosslinked with different glyoxal concentrations.

To evaluate the degradation process of the CS-GE hydrogels under collagenase treatment, the remaining weight of hydrogels was measured. The 0.0025% and 0.005% glyoxal-crosslinked hydrogels were degraded within 8 days, and it took 12 days to fully degrade the 0.01% glyoxal-crosslinked hydrogel (Figure 5A).

### 2.7. Release Pattern of FITC-Dextran from Hydrogels

We assessed the release profiles of FITC-dextran of different molecular weights (4, 40 and 500 kDa) from the CS-GE hydrogels crosslinked with different concentrations of glyoxal (0.0025%, 0.005% and 0.01%). When encapsulated with 4 kDa FITC-dextran, all hydrogel groups displayed a similar release profile. At 12 h, the cumulative release from hydrogels crosslinked with 0.0025%, 0.005% and 0.01% glyoxal were 43.4% ± 2.0%, 39.2% ± 2.3% and 25.5% ± 8.3%, respectively. After 72 h, nearly 90% of dextran had been released in all the experimental groups (Figure 5B). When encapsulated with 40 kDa FITC-dextran, the cumulative release from hydrogels crosslinked with 0.0025%, 0.005% and 0.01% glyoxal were 58.2% ± 5.2%, 53.7% ± 7.6% and 48.1% ± 4.8%, respectively, at 12 h. Similarly, almost 90% of dextran had been released at 72 h (Figure 5C). When encapsulated with 500 kDa FITC-dextran, the cumulative release from the CS-GE hydrogels crosslinked with 0.0025%, 0.005% and 0.01% glyoxal were 40.6% ± 6.4%, 36.8% ± 1.5% and 36.8% ± 1.5%, respectively, at 12 h. At 72 h, the cumulative release of the 0.0025% and 0.005% glyoxal were nearly 80%, but that of the 0.01% glyoxal group was less than 65% (Figure 5D). Since no apparent difference was noted between the 0.0025% and 0.005% glyoxal groups, a glyoxal concentration of 0.005%, which led to better biomechanical characteristics, was chosen for the following experiments.

### 2.8. HPL-Incorporated Hydrogel Enhanced the Cell Performance

**Figure 6:**
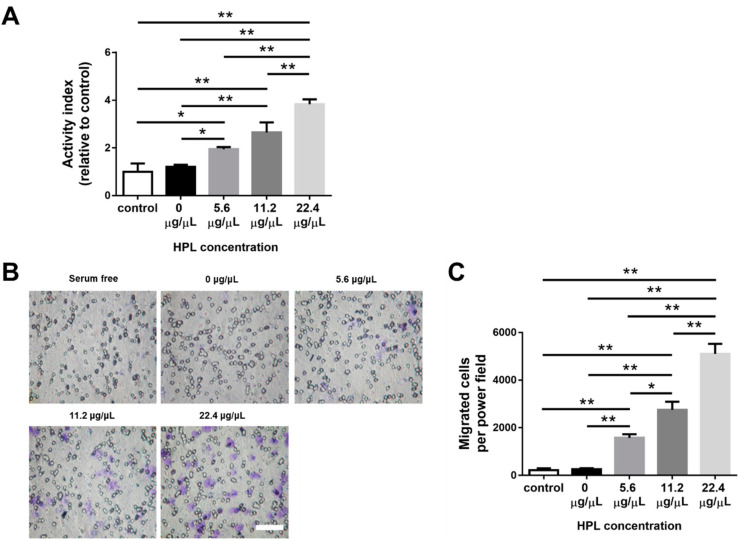
Effect of HPL-incorporated CS-GE hydrogels on the proliferation of Hs68 fibroblasts and the migration of HUVECs. (**A**) Alamar Blue assay revealed the higher content of HPL in the hydrogel facilitated the proliferative activity of Hs68 fibroblasts better relative to serum free group on day 5. (**B**) The representative images of HUVEC migration with different concentrations of HPL-incorporated hydrogel (scale bar = 100 μm). (**C**) Migration of endothelial cells toward CS-GE hydrogels was significantly enhanced by a higher content of HPL in the hydrogel. (* *p* < 0.05, ** *p* < 0.01).

The effect of HPL-incorporated CS-GE hydrogel on Hs68 cells was evaluated by Alamar Blue assay. The relative proliferative activity of the 22.4 μg/μL HPL group on day 5 was significantly higher than that of all the other groups (22.4 μg/μL HPL: 3.8 ± 0.6-fold vs. 0 μg/μL HPL: 1.2 ± 0.3-fold, 5.6 μg/μL HPL: 1.9 ± 0.3-fold and 11.2 μg/μL HPL: 2.6 ± 1.2-fold from control, *p* < 0.01, respectively; Figure 6A). To evaluate the effect of HPL-incorporated hydrogels on the migration of HUVECs, we adopted a transwell model with HPL-encapsulated hydrogels preloaded in the lower well. HUVECs were seeded in the transwells, and we counted the cell number that migrated toward the hydrogel-loaded lower wells. The cells on the undersurface of the transwell membrane were stained with crystal violet at 24 h (Figure 6B). As the HPL content in the hydrogel increased, more cell migration was noted. The number of migrant cells in the 22.4 μg/μL HPL group was significantly higher than that in all the other groups (22.4 μg/μL HPL: 5103.2 ± 940.1 cells per power field vs. control group: 221.6 ± 168.9, 0 μg/μL HPL: 255.4 ± 109.5, 5.6 μg/μL HPL: 1575.2 ± 340.9 and 11.2 μg/μL HPL: 2753.2 ± 759.7 cells per power field, *p* < 0.01, respectively; Figure 6C).

### 2.9. Growth Factor Release from HPL-Incorporated Hydrogel

**Figure 7:**
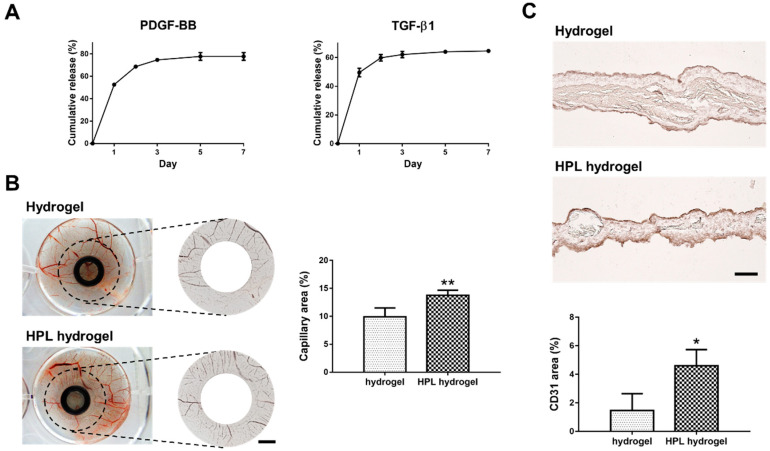
The release of growth factors from CS-GE hydrogels and chick chorioallantoic membrane (CAM) assay. (**A**) The cumulative releases of PDGF-BB and TGF-β1 from HPL-incorporated hydrogel. (**B**) The representative photographs of CAMs after treatment with CS-GE hydrogel only or HPL-incorporated CS-GE hydrogel, which were loaded on the CAMs of day 7 chick embryos. After 72 h of incubation, CAMs were excised and photographed (scale bar = 3 mm). Blood vessel formation on the CAM was quantified by measuring the capillary area percentage around the implanted hydrogels. (**C**) Immunohistochemistry staining of the endothelial marker CD31 in the CAM sections (scale bar = 100 μm). The ratio of the CD31-positive area was significantly larger in the group with HPL-incorporated CS-GE hydrogel. (* *p* < 0.05, ** *p* < 0.01).

The major mitogenic growth factors identified in HPL are platelet-derived growth factor-BB (PDGF-BB, ~25 kDa) and transforming growth factor-β1 (TGF-β1, ~44 kDa) [8]. Hence, we measured the concentration of these two growth factors in HPL by enzyme-linked immunosorbent assay (ELISA). The concentration of PDGF-BB was 9452.1 ± 696.1 pg/mL and that of TGF-β1 was 136,180.0 ± 6081.4 pg/mL. After incubation in PBS at 37 °C for 7 days, the cumulative releases of PDGF-BB and TGF-β1 from HPL-incorporated hydrogel were 77.7% ± 3.5% and 64.6% ± 1.0%, respectively (Figure 7A).

### 2.10. HPL-Incorporated Hydrogel Enhanced Angiogenesis In Ovo

The angiogenesis potential of HPL-incorporated CS-GE hydrogel was assessed by examining the capillary formation and the CD31 positive signal in chick chorioallantoic membrane (CAM) assay. After applying the hydrogels on CAM for 3 days, images of blood vessels around the samples were analyzed. Significantly higher capillary density was observed in the HPL-incorporated hydrogel group (capillary area: 13.8% ± 0.9% vs. 9.9% ± 1.6% of the hydrogel group, *p* < 0.01; Figure 7B). Moreover, a significantly higher ratio of CD31-positive area in the CAM sections of the HPL-incorporated hydrogel group was observed by immunohistochemistry (CD31-positive area: 4.6% ± 1.1% vs. 1.5% ± 1.2% of the hydrogel group, *p* < 0.05; Figure 7C)

## 3. Discussion

HPL contains lots of well-known platelet growth factors, which have been considered to play an essential role in cell-mediated angiogenesis and skin fibroblast behavior [33,34]. Previous publications have shown that HPL can be utilized for the restoration of corneal lesions [35], diabetic ulcers [36] and other soft tissue pathologies [37]. Apart from the soft tissue, HPL can be successfully applied for the local treatment of osteoarthritis, which is attributed to the growth factors released from platelet granules after intra-articular activation [38]. Mesenchymal stem cells can also be combined with HPL for bone regeneration [39]. However, HPL-based treatment may be limited because of the poor retention of HPL in the defect sites. In the present study, HPL-incorporated CS-GE hydrogel was successfully fabricated, and its mechanical property was improved by crosslinking with glyoxal. The HPL-incorporated CS-GE hydrogel not only significantly promoted the proliferative activity of Hs68 fibroblasts and the migration of HUVECs in vitro but also enhanced angiogenesis in the in ovo CAM model. Hence, the novel crosslinked hydrogel system allows sustained release of HPL-derived growth factors to promote tissue regeneration.

Chitosan-based hydrogels are often combined with β-GP to constitute a thermally responsive hydrogel system. Due to the electrostatic attractions between the chitosan protonated amine groups (-NH_3_^+^) and negatively charged phosphate molecules of β-GP (-HPO_4_^−^ or -PO_4_^2−^), the hydrogel system composed of chitosan, gelatin and β-GP can stay in a liquid state without aggregation at low temperature [40,41]. By adding β-GP as a weak base, the pH of the solution is escalated close to physiological pH so that the available -NH_2_ on the chitosan and gelatin chains may increase [42]. Therefore, the more available reaction sites can be bound by adding glyoxal as a crosslinker to form a robust network structure. Consequently, a higher glyoxal concentration led to greater mechanical strength and a shorter gelation time (less than ~70 s) in this study.

Recently, glyoxal has been used as an alternative dialdehyde crosslinker in various biomedical applications [42,43]. Although a higher amount of glyoxal was reported to trigger oxidative stress, thereby inducing cytotoxicity [44], we demonstrated no significant cytotoxicity if the concentration of glyoxal was less than 0.01% in cell culture. Compared to other chemical crosslinkers for CS-GE hydrogels, glyoxal exhibits several advantages for its application in tissue engineering and drug delivery. Genipin-crosslinked chitosan-based hydrogels were shown to have good biocompatibility and low cytotoxicity, but the gelation time was at least 1 h, rendering it not suitable for clinical use [45,46]. Moreover, an anti-angiogenesis effect of genipin was reported to decrease the rate of wound healing [47]. Glutaraldehyde can crosslink chitosan chains to form hydrogels within 1 h; however, it is considered toxic for the respiratory tract, eyes and skin [48].

Our previous study manifested blending gelatin in the chitosan-based hydrogel, resulting in a more porous architecture after in vivo application due to the degradation of gelatin [26]. This porous structure can ensure that more growth factors are released rather than stuck in the hydrogel. By modifying this design in the present study, the chemical crosslinker, glyoxal, was used to increase the mechanical strength of the hydrogel, rendering a more sustained release of the encapsulated HPL. Lim et al. fabricated HPL-incorporated hydroxybutyl chitosan hydrogels, which displayed a cumulative release of more than 80% of PDGF-BB and TGF-β1 on day 7 during in vitro degradation [49]. Compared to their result, the cumulative releases of PDGF-BB and TGF-β1 in our experiment were less than 80%, demonstrating a more sustained release of HPL.

In this study, a significantly higher cell proliferative activity was noted as the HPL concentration increased in the medium. In line with the previous publications suggesting that HPL is more suitable than FBS as culture supplements [50,51], our results revealed that 5% HPL was significantly more effective than 10% FBS to promote Hs68 fibroblast proliferation and migration. Li et al. have elucidated the mechanism of human dermal fibroblast migration driven by PDGF-BB, indicating the important role of PDGF in regulating the fibroblast behavior [52]. HPL exhibits abundant PDGF-BB, indicating the great potential of HPL in fibroblast-driven soft tissue regeneration, such as wound healing.

Tissue regeneration normally needs the formation of vessels to transfer essential materials including oxygen and nutrition. Therefore, capillary angiogenesis within the damaged tissue is critical during the tissue repair process. Angiogenesis is a coordinated process, involving endothelial cellular proliferation, migration and association in tubular structures [53]. In this work, HUVECs cultured in the medium containing 5% HPL revealed significantly more tube-like structures relative to the 10% FBS group, and the HPL-incorporated hydrogel significantly induced the migration of HUVECs. Moreover, the CAM assay was employed for further evaluation because it can be applied to study in ovo both angiogenesis and anti-angiogenesis in response to different materials [54].

In the result of the CAM assay, the capillary area percentage around the HPL-incorporated hydrogel was estimated to be significantly higher relative to that in the control group. Moreover, a significantly higher positive signal of CD31 can be observed in the HPL-incorporated hydrogel. These results suggested the potential of this HPL-incorporated hydrogel in promoting angiogenesis. Although the CAM assay is recognized as a valuable method to study the angiogenic activity of biomaterials, further investigation in the animal model is required. The results of this study demonstrated a promising approach to the fabrication of HPL-incorporated CS-GE hydrogel for tissue regeneration. Further animal studies are required to fully extrapolate the potential of this novel hydrogel system in clinical application.

## 4. Materials and Methods

### 4.1. Culture of Hs68 Cells and HUVECs

Hs68 cells (ATCC, Manassas, VA, USA) were cultured in Dulbecco’s modified Eagle’s medium—high glucose (DMEM-HG; HyClone, Logan, UT, USA) supplemented with 10% FBS (HyClone) and 1% penicillin/streptomycin (PS; Biological Industries, Cromwell, CT, USA). HUVECs (Lonza, Bend, OR, USA) were cultured in endothelial basal medium (EBM; Lonza) supplemented with 10% FBS and 1% PS. The cells were cultured in a 5% CO_2_ humidified atmosphere at 37 °C, and the medium was refreshed every 2–3 days. Upon 90% confluence, the cells were washed with PBS (Omics Biotechnology, New Taipei City, Taiwan) and detached using trypsin-ethylenediaminetetraacetic acid (EDTA) (Biological Industries) for various experiments.

### 4.2. The Effect of HPL on Cell Proliferation

Commercially available HPL (UltraGRO^TM^; Helios, Sarasota, FL, USA) was used for cell culture supplements. Hs68 cells and HUVECs were seeded in a 24-well culture plate at densities of 1.5 × 10^4^ and 1 × 10^4^ cells/well, respectively. After culturing for 24 h, the media were changed to media supplemented with 1% HPL, 2% HPL, 5% HPL or 10% FBS. For each group, 0.5 mL medium containing 10% (*v*/*v*) Alamar blue reagent (Invitrogen, Waltham, MA, USA) was added and incubated in 5% CO_2_, 37 °C for 2 h on days 1, 3 and 5. The fluorescence signals at an excitation wavelength of 560 nm and an emission wavelength of 590 nm were measured using a standard spectrophotometer (Tecan Trading AG, Männedorf, Switzerland).

### 4.3. In Vitro Wound Healing Assay with Hs68 Cells

An in vitro wound healing assay of Hs68 cells was performed using cell culture inserts (Corning, Corning, NY, USA) to create a cell-free area of the same size in each well. Hs68 cells were seeded into a 24-well culture plate with a small rectangular culture insert placed at the center of each well. The seeding density was 2 × 10^4^ cells/well, and the cells were incubated in DMEM-HG supplemented with 10% FBS and 1% PS for 4 h to allow attachment. Subsequently, the inserts were removed, creating a rectangular cell-free zone in each well. After washing twice with PBS, the medium was changed to DMEM-HG supplemented with 1% HPL, 2% HPL, 5% HPL or 10% FBS. Serum-free DEME-HG was used as a control group. The migration of Hs68 cells into the cell-free zone was observed using a time-lapse microscope (DMI 6000, Leica, Wetzlar, Germany), and ImageJ software (NIH, Bethesda, MD, USA) was used to quantify the wound closure area of Hs68 fibroblasts at 12 h.

### 4.4. In Vitro Tube Formation Assay with HUVECs

HUVECs were seeded on μ-slides (Ibidi, Fitchburg, WI, USA) coated with Matrigel (Corning) at a density of 6 × 10^4^ cells/well, and they were cultured with EBM supplemented with 1% HPL, 2% HPL, 5% HPL or 10% FBS. Serum-free EBM was used as a control group. Tube-like structures were observed and captured with a phase-contrast microscope at 2, 4 and 6 h. Analysis of microscopic images at 4 h was proceeded by the plug-in Angiogenesis Analyzer in ImageJ software.

### 4.5. Preparation of Crosslinked CS-GE Hydrogels

The CS-GE hydrogel was prepared by a protocol modified from previous reports [26,54]. Briefly, 4% chitosan (chitosan glutamate salt, molecular weight: 200–600 kDa, degree of deacetylation: 75–90% *w*/*v*, in ddH_2_O, NovaMatrix, Sandvika, Norway) and 10% gelatin (*w*/*v*, in ddH_2_O, St. Louis, MO, USA) were prepared at 60 °C and sterilized by autoclaving. β-GP (28.5% *w*/*v*, in ddH_2_O, Sigma) was prepared and filter sterilized. After 0.4 mL of 10% gelatin solution was blended with 2 mL of 4% chitosan solution and 0.6 mL ddH_2_O homogeneously, 1 mL 28.5% β-GP solution was added drop-wise into the chitosan/gelatin solution with stirring. The CS-GE mixture was composed of 2% chitosan, 1% gelatin and 7.125% β-GP. The glyoxal solution was diluted with ddH_2_O until the concentration reached 0.4%. A different volume of 0.4% glyoxal (Sigma) was subsequently added into the CS-GE mixture to reach a final concentration of glyoxal ranging from 0.0025% to 0.04% in the hydrogel.

### 4.6. Gelation Time and Rheological Studies

To assess the gelation time, 1 mL of CS-GE mixture crosslinked with 0.0025%, 0.005%, 0.01%, 0.02% and 0.04% glyoxal was loaded into a 15 mL centrifuge tube and incubated at 37 °C. Gelation due to the crosslinking between the amine groups in chitosan and gelatin molecules was estimated by the vial tilting method [55].

To determine the linear viscoelastic properties of the CS-GE hydrogels, small-amplitude oscillatory shear experiments were performed using a modular compact rheometer (Discovery Hybrid Rheometer, HR 20, TA instruments, New Castle, DE, USA) equipped with a stainless-steel parallel-plate geometry (20 mm in diameter) and an electrically heated plate. CS-GE hydrogels crosslinked with 0.0025%, 0.005%, 0.01%, 0.02% and 0.04% glyoxal were shaped into 20 mm in diameter and 5 mm in height. The frequency sweep test was performed over a frequency ranging from 0.01 to 10 Hz at 37 °C, and the strain was set at 1% to record the storage modulus (G′) and loss modulus (G″).

### 4.7. Swelling Test

To examine the fluid-retaining capability of the hydrogels, 1 mL of CS-GE mixture crosslinked with 0.0025%, 0.005%, 0.01%, 0.02% and 0.04% glyoxal was loaded into a 15 mL centrifuge tube. After full gelation, the sample was frozen at −20 °C and lyophilized. Dried hydrogels were then incubated in PBS at 37 °C. The wet weight of each sample was measured at several time points during the incubation. The swelling ratio of hydrogels was calculated by the following equation (W_s_ = swollen hydrogel weight at different time points, W_d_ = dried hydrogel weight):

$$\mathrm{Swelling}\;\mathrm{ratio}\;(\%)\;=\;\frac{W_{s}\;-\;W_{d}}{W_{d}}\;\times \;100\%$$

### 4.8. Cytotoxicity Study of the Crosslinked Hydrogels

CS-GE hydrogels crosslinked with 0%, 0.0025%, 0.005% and 0.01% glyoxal (100 μL) were added into 12 transwell inserts (Corning) with 8.0 μm pore size. Subsequently, the transwell inserts were put into a 12-well plate, which was previously seeded with Hs68 at a density of 30,000 cells/well. The cell viability was quantified by Alamar Blue assay on days 1, 3 and 5.

### 4.9. Scanning Electron Microscopy

CS-GE hydrogels were crosslinked with different concentrations of glyoxal, frozen at −20 °C and lyophilized. Samples were then sectioned, sputter-coated with platinum and examined using an SEM (Nova^TM^ NanoSEM 23, FEI, Hillsboro, OR, USA). The pore size of crosslinked hydrogels was estimated by tracing the periphery of the pores using ImageJ software. We selected the pores with a long-to-short axis ratio of not more than 1.5 for measurements, and at least 30 pores for each sample were estimated. 

### 4.10. In Vitro Enzymatic Degradation Test

The in vitro degradation test was performed by immersing the crosslinked hydrogels in PBS containing 25 U/mL collagenase type I (Gibco, Waltham, MA, USA) at 37 °C, and the samples were weighed at different time points. The remaining weight of hydrogel was determined by the following equation (W_i_ = initial weight, W_x_ = weight at different time points):

$$\mathrm{Remaining}\;\mathrm{weight}\;\mathrm{of}\;\mathrm{hydrogel}\;(\%)\;=\;\frac{W_{x}}{W_{i}}\;\times \;100\%$$

### 4.11. FITC-Dextran Release

To determine the release pattern of particles with different molecular weights from the crosslinked hydrogels, we modified a dextran release experiment from a previous study [56]. Briefly, the CS-GE mixture blended with three different molecular weights of FITC-dextrans (4, 40 and 500 kDa; Sigma) were crosslinked with 0.0025%, 0.005% and 0.01% glyoxal, followed by the incubation in PBS at 37 °C. At 12, 24, 48 and 72 h, PBS was collected, and then new PBS was added. FITC-dextran released into PBS from hydrogels was measured by a Spark™ 10M multimode microplate reader (Tecan Trading AG, Männedorf, Switzerland).

### 4.12. HPL-Incorporated Hydrogel Enhanced Hs68 Cell Proliferation

CS-GE hydrogels blended with different concentrations of HPL (0, 5.6, 11.2 and 22.4 μg/μL) were crosslinked with 0.005% glyoxal, and 100 μL of the mixture was added into 24 transwell inserts with 8.0 μm pore size. The transwell inserts were put into a 24-well plate previously seeded with Hs68 cells at a density of 30,000 cells/well. Serum-free DEME-HG was used for cell culture, and the control group exhibited no hydrogel. The fluorescence signal of the Alamar Blue assay on day 5 was quantified as an activity index by normalizing it to the control group.

### 4.13. HPL-Incorporated Hydrogel Enhanced HUVEC Migration

CS-GE hydrogels blended with different concentrations of HPL (0, 5.6, 11.2 and 22.4 μg/μL) were crosslinked with 0.005% glyoxal, and 250 μL of the mixture was added into each well of a 24-well cell culture plate and immersed in 700 μL of serum-free EBM. Subsequently, transwell inserts with 8.0 μm pore size were placed into each well, and 100 μL of serum-free EBM containing 5 × 10^4^ HUVECs was added into each insert. After incubation for 24 h, all nonmigrant cells were removed from the upper surface of the transwell membrane using a cotton swab, and migrant cells at the undersurface were fixed and stained with crystal violet (Sigma). The control group exhibited no hydrogel. Images were obtained, and the number of cells stained with crystal violet was counted by ImageJ software. Values were reported as the number of cells migrated per power field.

### 4.14. In Vitro Growth Factor Release into the Medium

The release of growth factors from HPL-incorporated hydrogels was assessed by ELISA. Briefly, 250 μL of CS-GE mixture blended with 22.4 μg/μL HPL and crosslinked with 0.005% glyoxal was loaded into each well of a 24-well culture plate, followed by immersion in 250 μL of PBS at 37 °C. On days 1, 2, 3, 5 and 7, the PBS was collected and replaced with 250 μL of new PBS. The concentration of PDGF-BB and TGF-β1 preserved in PBS was determined using the relevant ELISA kits (R&D Systems, Minneapolis, MN, USA).

### 4.15. Angiogenesis Assay in the CAM Model

Fertilized chicken eggs were obtained from the Animal Health Research Institute of Taiwan. Eggs were incubated under constant 80% humidity at 37 °C in a egg hatching machine (Rcommax50, Rcom, Kimhae, Korea). On day 3, an open window was made on the air chamber, and the embryo viability was evaluated. On day 7, we placed an 8 mm o-ring onto the CAM, and 100 μL of crosslinked CS-GE hydrogel blended with 22.4 μg/μL HPL was applied into the o-ring. The window on the shell was then sealed with Tegaderm (3M, Maplewood, MN, USA) to prevent dehydration and contamination. On day 10, the embryos were infused with 4% paraformaldehyde and placed at −80 °C overnight. The o-ring and adjacent CAM tissue were carefully removed and transferred to a 6-well culture plate containing 4% paraformaldehyde. Subsequently, the CAM specimens were photographed, and a circular area of 2 cm around the o-ring was marked, and the percentage of the capillary area in this donut area was analyzed by ImageJ.

For the immunohistochemical staining of the CAM sections, the CAM specimens were embedded into paraffin, sectioned (5 μm thick) and stored at room temperature until use. Briefly, the sections were dewaxed by incubation in xylene substitute (Leica) and hydrated in a series of graded ethanol solutions. Antigen retrieval was performed using pH 9.0 Tris-EDTA solution for 30 min at 95 °C. Then, the sections were treated with 0.3% H_2_O_2_ for 10 min and incubated with anti-CD31 (Abcam, ab28364, Cambridge, England) at 4 °C overnight. Secondary horseradish peroxidase (HRP) antibodies were applied, and the sections were incubated with diaminobenzidine (DAB; BioTnA, Kaohsiung, Taiwan) for 2 min. Negative controls without utilizing primary antibodies were used to rule out nonspecific binding. The CAM sections were observed using a microscope, and the CD31-positive area was quantified by the ImageJ software.

### 4.16. Statistical Analysis

All measurements are presented as the means ± standard deviation. The data were analyzed using an independent-sample Student’s t-test or one-way ANOVA with Tukey’s post hoc test. All statistical analyses were performed using GraphPad Prism 7 (La Jolla, San Diego, CA, USA), and statistically significant differences were defined as *p* < 0.05.

## 5. Conclusions

In this study, CS-GE hydrogels crosslinked with different concentrations of glyoxal were fabricated. The crosslinking degree substantially affected the fluid-retaining ability and degradability of the hydrogels. HPL-encapsulated hydrogel significantly improved Hs68 fibroblast proliferation and HUVEC migration during in vitro culture. Moreover, in vitro degradation assay and growth factor release assay showed that crosslinked CS-GE hydrogel can serve as a carrier for the sustained release of HPL. In the CAM assay, the HPL-incorporated hydrogel significantly enhanced angiogenesis relative to the hydrogel group, indicating a great potential in promoting angiogenesis. Although further animal studies are required to fully extrapolate its clinical potential, the results of this study demonstrated a promising approach to the fabrication of HPL-encapsulated CS-GE hydrogels for tissue regeneration.

## Figures and Tables

**Table 1:** Gelation time of CS-GE hydrogel crosslinked with different glyoxal concentrations.

Glyoxal (%)	0.0025%	0.005%	0.01%	0.02%	0.04%
Gelation time (s)	63.0 ± 7.1	39.7 ± 2.9	21.0 ± 1.7	15.7 ± 3.8	13.7 ± 1.5

## Data Availability

Data available in a publicly accessible repository.
